# Challenges Associated With Diverticular Bleeding in an Elderly Female Patient With Situs Inversus Totalis: A Case Report and Literature Review

**DOI:** 10.7759/cureus.73410

**Published:** 2024-11-10

**Authors:** Tade Popovic, Goran Bokan, Ante Bogut, Kristian Karlovic

**Affiliations:** 1 Department of Nephrology, Internal Medicine Clinic Mostar, Faculty of Medicine, University Clinical Hospital Mostar, Mostar, BIH; 2 Department of Gastroenterology, Internal Medicine Clinic, Faculty of Medicine, University Clinical Centre of the Republic of Srpska, Banja Luka, BIH; 3 Department of Gastroenterology, Internal Medicine Clinic Mostar, Faculty of Medicine, University Clinical Hospital Mostar, Mostar, BIH; 4 Department of Radiology, Faculty of Medicine, University Clinical Hospital Mostar, Mostar, BIH

**Keywords:** comorbidities, diverticular bleeding, diverticulitis, older female patient, situs inversus totalis

## Abstract

Diverticulitis is a relatively common condition in gastroenterology. It is most often presented by inflammation of one or more diverticula, depending on their presence in the colon, and by abdominal pain and bleeding. Bleeding from the diverticulum has a wide range of clinical manifestations, which in a certain percentage of cases can have a very unfavorable course and prognosis. Situs inversus totalis (SIT) is a congenital condition with multiple, predominantly positional abnormalities of the organs in relation to their anatomical positions. Unlike bleeding from the diverticulum, SIT occurs very rarely, and cases of diverticulitis and accompanying bleeding from the diverticulum in SIT are extremely rare; there are only a handful of cases described in the literature. In this study, we present the diagnostic challenge of identifying and treating diverticulitis in an elderly patient with SIT and accompanying chronic comorbidities.

## Introduction

Situs inversus totalis (SIT) is a rare congenital condition in which the major visceral organs are completely mirror-image transposed along the left-right axis. It usually occurs in one in 8,000-25,000 live births, while the frequency of occurrence in the adult population is still unknown and is based on assumptions. As a multifactorial disease, genetic factors, environmental factors, and developmental stochasticity are responsible for the occurrence of SIT [1].

Several different forms of this malformation are described in the literature, the most common of which is situs inversus totalis, with a frequency of 82.7%. The most frequent abdominal manifestation of SIT is cholelithiasis, which, according to the literature, occurs in about one in 10 patients, while the frequency of diverticulitis is very low, with limited data available in the literature [2].

Diverticulitis refers to the inflammation of the diverticula, often accompanied by bleeding and either microscopic or macroscopic perforations, which usually require conservative treatment and, in complicated cases, may require surgical intervention [3]. After searching the PubMed database for the terms "situs inversus totalis" and "diverticulitis," seven papers were found, of which three met our criteria for comparison.

## Case presentation

**Figure 1:**
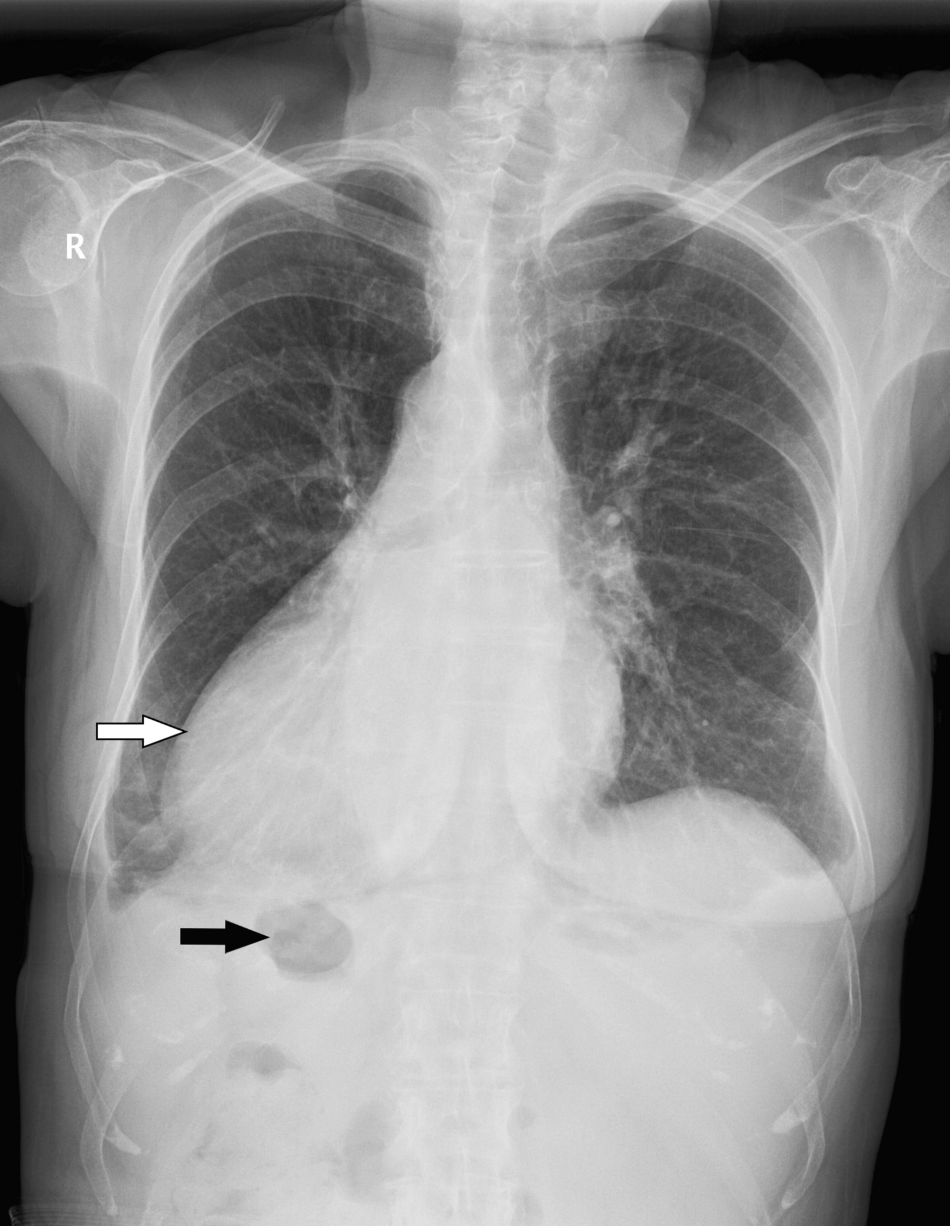
X-ray of the heart and lungs showing the upper part of the abdomen. Dextrocardia (white arrow) and the stomach (black arrow) are visualized on the right side. There are no signs of pneumoperitoneum.

A 73-year-old female patient was hospitalized due to intense abdominal pain, anamnestic data on elevated body temperature of up to 39°C, and bleeding from the lower parts of the gastrointestinal tract. The symptoms first appeared four days before hospitalization. Abdominal pains were of a diffuse nature, but on the date of the examination, they were localized in the upper right quadrant of the abdomen and were described as stabbing. Three days before hospitalization, the patient had a fever of up to 39°C, as well as a black stool, while on the date of the hospitalization, she noticed fresh blood in the stool. During the examination, she was afebrile and slightly hypertensive 150/70 mmHg, the abdomen was painful to touch in the upper right quadrant, but without signs of tightening stomach muscles. Traces of dark stool and admixture of fresh blood were observed during the digital rectal examination (DRE). The remaining physical medical results were normal. From the medical history, we learned that 10 years ago she had an operation on her right hip (total endoprosthesis) and that she suffered from hypertension. An ultrasonographic examination of the abdomen was performed that revealed normal results in the solid intra-abdominal organs and ruled out the possibility of cholecystitis as the cause of pain in the upper right quadrant. No signs of pneumoperitoneum were found in the X-ray of the heart and lungs and the native abdomen (Figure 1). We subsequently received information about SIT from the patient. Emergency multi-slice computed tomography (MSCT) visualizes multiple diverticula with signs of diverticulitis. Laboratory and biochemical analyses of the sampled blood are presented in Table 1 and Table 2, in which the significantly elevated values ​​of leukocytes and CRP stand out.

**Table 1:** Monitoring the trend of laboratory and biochemical indicators during the hospitalization.

Parameters	Upon admission	Day 1	Day 2	Day 6	Reference range
White blood cell	30.6×10^9^/L	31.2×10^9^/L	22.1×10^9^/L	9.6×10^9^/L	3.4-9.7×10^9^/L
C-reactive protein	120 mg/L	118 mg/L	113.2 mg/L	11 mg/L	0.0-5.0 mg/L
Red blood cells	5.22×10^12^/L	4.80×10^12^/L	4.32×10^12^/L	4.56×10^12^/L	3.86-5.08×10^12^/L
Hemoglobin	154 g/L	140 g/L	128 g/L	134 g/L	119-157 g/L
Hematocrit	0.434 L/L	0.428 L/L	0.365 L/L	0.380 L/L	0.356-0.470 L/L
Mean corpuscular volume	83.1 fL	89.3 fL	84.5 fL	83.3 fL	83.0-97.2 fL
Blood platelet	354×10^9^/L	326×10^9^/L	294×10^9^/L	303×10^9^/L	158-425×10^9^/L
Glucose	6.2 mmol/L	5.9 mmol/L	5.0 mmol/L	6.0 mmol/L	4.4-6.4 mmol/L
Sodium	139 mmol/L	143 mmol/L	140 mmol/L	143 mmol/L	137-146 mmol/L
Potassium	3.9 mmol/L	2.7 mmol/L	3.5 mmol/L	3.9 mmol/L	3.9-5.1 mmol/L
Chlorine	107 mmol/L	105 mmol/L	106 mmol/L	108 mmol/L	97-108 mmo/L

**Table 2:** Laboratory results on admission.

Parameters	Upon admission	Reference range
Aspartate aminotransferase	15 IU/L	8-30 IU/L
Alanine aminotransferase	12 IU/L	10-36 IU/L
Alkaline phosphatase	123 IU/L	54-119 IU/L
γ-glutamyltransferase	45 IU/L	9-35 IU/L
Lactate dehydrogenase	245 IU/L	124-241 IU/L
Creatine kinase	29 IU/L	0-153 IU/L

**Figure 2:**
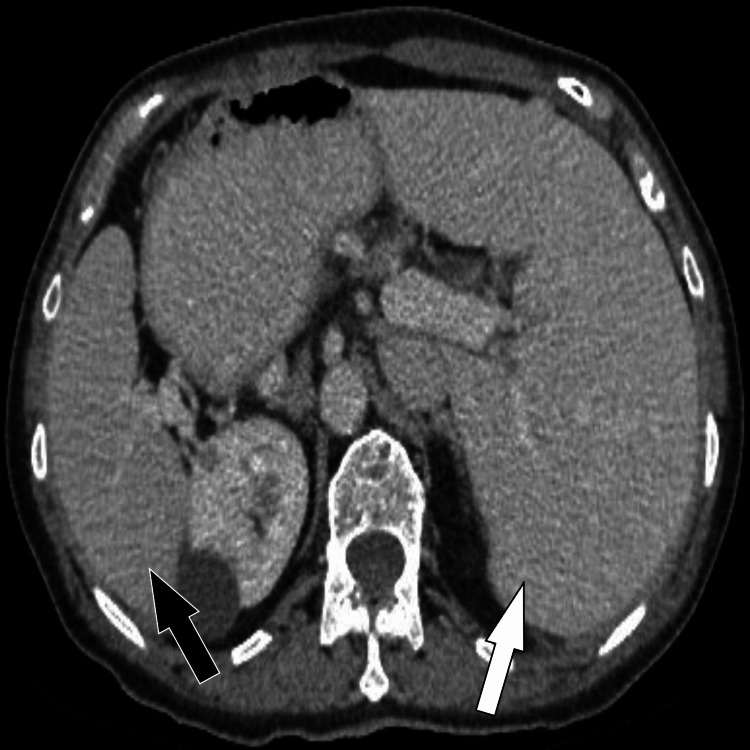
MSCT axial section (venous phase) through the upper part of the abdomen shows the liver on the left side (white arrow) and spleen on the right side (black arrow). MSCT: multi-slice computed tomography

**Figure 3:**
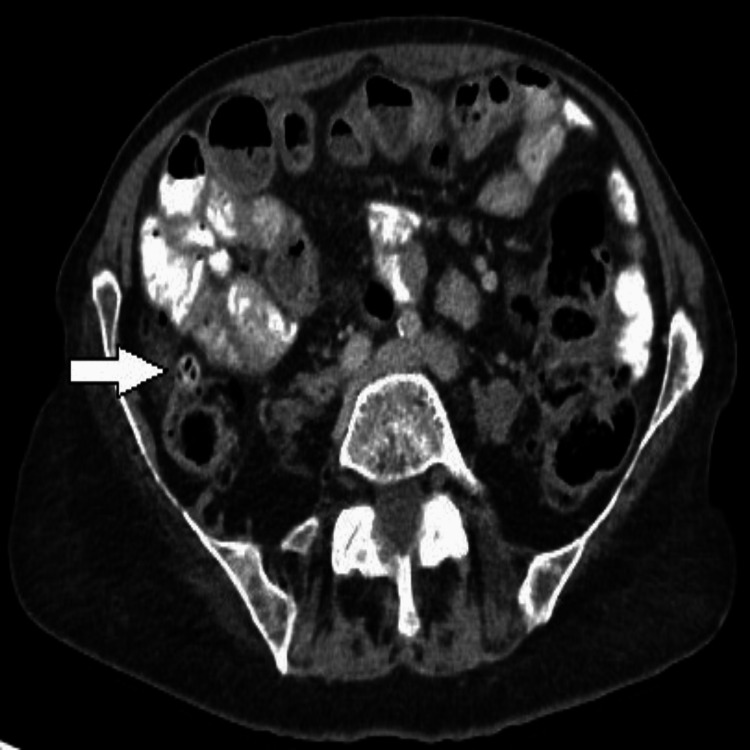
MSCT axial section (venous phase) through the lower part of the abdomen shows the diverticulum indicated by the arrowhead. MSCT: multi-slice computed tomography

**Figure 4:**
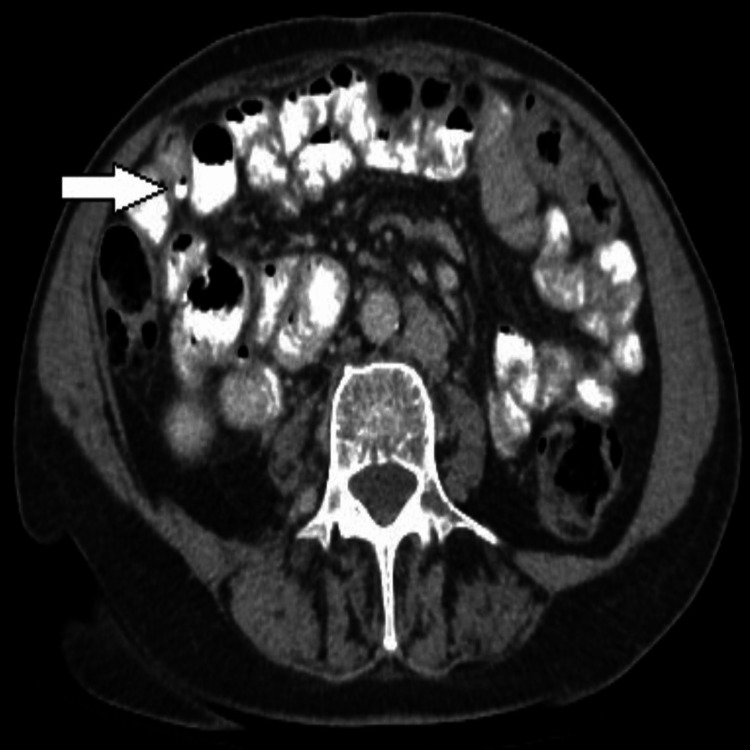
MSCT axial section (venous phase) through the lower part of the abdomen shows the diverticulum indicated by the arrowhead. MSCT: multi-slice computed tomography

After an MSCT of the abdomen and pelvis, SIT is observed. Figure 2 shows the mirror-image transposition of the liver and spleen, and Figures 3, 4 show the diverticula. No signs of pneumoperitoneum.

On the first day after admission, a paroxysm of atrial fibrillation occurred, which was converted to sinus rhythm after administering the prescribed amiodarone therapy. The patient was treated with dual antibiotics (ciprofloxacin and metronidazole), probiotics, proton pump inhibitors, calcium channel blockers, as well as crystalloid solutions. On the sixth day of the hospitalization, there was improvement in clinical and laboratory tests, and the patient was discharged for further treatment at home. Two months after the treatment at the hospital, a total colonoscopy was performed, which visualized numerous diverticula proximal to the sigmoid colon without signs of inflammation.

## Discussion

Situs inversus totalis (SIT) is a rare congenital condition of multi-factorial etiology in which the major visceral organs are completely mirror-image transposed. Information about the diagnosis of SIT at different ages in the literature is rather scarce [1]. Comorbidities are also poorly documented, and information on the frequency of diverticulitis in patients with SIT was unavailable [2,3]. More than 50% of people over 60 years of age and over 60% of people over 80 years of age have diverticula. In patients with diverticulosis, the risk of developing diverticulitis during their lifetime is estimated at 10-20% [4]. The pathophysiology of diverticulitis is not fully understood. Long-standing but unproven theories suggest that diverticulitis results from obstruction and trauma to the diverticulum with subsequent ischemia, microperforation, and infection [5]. In this paper, the difference in the clinical presentation of diverticulitis in patients with SIT was especially emphasized, as well as the anamnesis and physical examination. The typical clinical picture includes patients often presenting with acute, constant abdominal pain that is usually in the lower left quadrant [6]. Other possible symptoms include anorexia, constipation, nausea, diarrhea, and dysuria. Patients may have a history of diverticulitis or diverticulosis. Patients with diverticulitis usually have a fever (below 39°C), with tachycardia and hypotension that can also be present. During the examination, tenderness manifested exclusively in the lower left quadrant significantly increases the likelihood of diverticulitis, as does a palpable mass and abdominal distension [6].

Complications of diverticulitis may vary and it can be difficult to diagnose diverticulitis as the underlying cause of severe complications. Small perforations, surrounding preserved walls are common during the disease and most cases can be treated conservatively with antibiotics and supportive medical treatment. However, uncommon and severe complications such as uncontrolled perforation, phlegmon and abscess, phlebitis, intestinal obstruction, bleeding, and fistula require intensive treatment [7]. In the example of the patient with SIT in her anamnesis, the clinical presentation of pain in the upper right quadrant of the abdomen stands out as a peculiarity.

The patient was treated with dual antibiotics (ciprofloxacin and metronidazole), probiotics, proton pump inhibitors (PPIs), calcium channel blockers, and crystalloid solutions. After two months, a follow-up colonoscopy was performed, identifying numerous diverticula without signs of inflammation. By typing the terms "situs inversus totalis and diverticulitis" in the PubMed index database, seven papers were found, three of which fulfilled our criteria for comparison.

In the paper by Mihetiu et al., a 44-year-old patient with pain in the lower right quadrant was presented, who had previously been unaware of SIT, and after anamnesis and physical examination, along with laboratory diagnostics, laparoscopic abdominal surgery was performed without radiological processing, during which SIT and diverticulitis were diagnosed. Postoperatively, after the MSCT of the abdomen, they additionally observed appendiceal agenesis. The patient was discharged for treatment at home three days after the admission [8].

Two studies by Karabay et al. and Sanjaj et al. presented the laparoscopic resection of the colon due to recurrent diverticulitis in a patient, highlighting the difficulties in planning and performing the operation itself. The authors provided differing opinions regarding the safety of laparoscopy as the method of choice for colon resection in patients with SIT [3,9].

A review article by Osarenkhoe concluded that there could be a significant connection between SIT and acquired comorbidities, such as cholelithiasis, colon carcinoma, stomach carcinoma, and lung carcinoma. It should be noted that diverticulosis/diverticulitis is not mentioned in the given article. We also note that within the systems of affected malformations in SIT, the digestive system predominates with 57.1% [2].

## Conclusions

The diagnostic knowledge that a patient has situs inversus totalis at any age represents an unusual and challenging condition, and to an even greater extent if there is a concomitant complex and acute pathology. Due to its low frequency, few available cases in the literature, and challenges in access and treatment, it represents a special challenge when making decisions about diagnostic and therapeutic interventions, which can often applied in the wrong order, and impose the need for increased caution.
